# Contrasting Spatial Distribution and Risk Factors for Past Infection with Scrub Typhus and Murine Typhus in Vientiane City, Lao PDR

**DOI:** 10.1371/journal.pntd.0000909

**Published:** 2010-12-07

**Authors:** Julie Vallée, Thaksinaporn Thaojaikong, Catrin E. Moore, Rattanaphone Phetsouvanh, Allen L. Richards, Marc Souris, Florence Fournet, Gérard Salem, Jean-Paul J. Gonzalez, Paul N. Newton

**Affiliations:** 1 Institut de Recherche pour le Développement, UMR190, Marseille, France; 2 Université Paris Ouest La Défense, Laboratoire Espace, Santé et Territoire, Nanterre, France; 3 Wellcome Trust-Mahosot Hospital-Oxford Tropical Medicine Research Collaboration, Mahosot Hospital, Vientiane, Lao PDR; 4 Nuffield Department of Clinical Medicine, Centre for Clinical Vaccinology and Tropical Medicine, University of Oxford, Churchill Hospital, Oxford, United Kingdom; 5 Rickettsial Diseases Research Program, Naval Medical Research Center, Silver Spring, Maryland, United States of America; 6 Faculty of Science, Center of Excellence for Vectors and Vector Borne Diseases, Mahidol University, Nakhon Pathom, Thailand; 7 Remote Sensing & Geographic Information Systems Field of Study (RS&GIS FoS), Asian Institute of Technology, Pathumthani, Thailand; 8 Centre International de Recherches Médicales de Franceville (CIRMF), Libreville, Gabon; University of Texas Medical Branch, United States of Ameica

## Abstract

**Background:**

The aetiological diagnostic of fevers in Laos remains difficult due to limited laboratory diagnostic facilities. However, it has recently become apparent that both scrub and murine typhus are common causes of previous undiagnosed fever. Epidemiological data suggests that scrub typhus would be more common in rural areas and murine typhus in urban areas, but there is very little recent information on factors involved in scrub and murine typhus transmission, especially where they are sympatric - as is the case in Vientiane, the capital of the Lao PDR.

**Methodology and Principal Findings:**

We therefore determined the frequency of IgG seropositivity against scrub typhus (*Orientia tsutsugamushi*) and murine typhus (*Rickettsia typhi*), as indices of prior exposure to these pathogens, in randomly selected adults in urban and peri-urban Vientiane City (n = 2,002, ≥35 years). Anti-scrub and murine typhus IgG were detected by ELISA assays using filter paper elutes. We validated the accuracy of ELISA of these elutes against ELISA using serum samples. The overall prevalence of scrub and murine typhus IgG antibodies was 20.3% and 20.6%, respectively. Scrub typhus seropositivity was significantly higher among adults living in the periphery (28.4%) than in the central zone (13.1%) of Vientiane. In contrast, seroprevalence of murine typhus IgG antibodies was significantly higher in the central zone (30.8%) as compared to the periphery (14.4%). In multivariate analysis, adults with a longer residence in Vientiane were at significant greater risk of past infection with murine typhus and at lower risk for scrub typhus. Those with no education, living on low incomes, living on plots of land with poor sanitary conditions, living in large households, and farmers were at higher risk of scrub typhus and those living in neighborhoods with high building density and close to markets were at greater risk for murine typhus and at lower risk of scrub typhus past infection.

**Conclusions:**

This study underscores the intense circulation of both scrub and murine typhus in Vientiane city and underlines difference in spatial distribution and risk factors involved in the transmission of these diseases.

## Author Summary

Scrub typhus and murine typhus are neglected but important treatable causes of fever, morbidity and mortality in South-East Asia. Epidemiological data suggests that scrub typhus would be more common in rural areas and murine typhus in urban areas but there are very few comparative data from places where both diseases occur, as is the case in Vientiane, the capital of the Lao PDR. We therefore determined the frequency of IgG antibody seropositivity against scrub typhus and murine typhus, as indices of prior exposure to these pathogens, in a randomly selected population of 2,002 adults living in different neighbourhoods in Vientiane. The overall prevalence of IgG against these two pathogens was ∼20%. However, within the city, the spatial distribution of IgG against these two diseases was radically different - past exposure to murine typhus being more frequent in urbanized areas while past exposure to scrub typhus more frequent in outlying areas. This study underscores the importance of ecological characteristics in improving the understanding of both scrub typhus and murine typhus transmission and epidemiology.

## Introduction

Scrub typhus and murine typhus are important but under-recognized treatable causes of fever, morbidity and mortality in South-East Asia [1], [2], [3], [4]. There has been a recent resurgence of interest in these diseases, which both cause undifferentiated fever, headache and myalgia progressing, in a minority, to jaundice, pneumonitis and meningo-encephalitis [5], [6], [7], [8], [9]. Scrub typhus, caused by *Orientia tsutsugamushi*, occurs in Asia and northern Australia and is transmitted by the bites of infected trombiculid mites [10]. Murine typhus, caused by *Rickettsia typhi*, occurs globally and is transmitted through the infected flea bite site or by scratching infected faeces into the skin [11], [12]. Scrub typhus and murine typhus were first differentiated, in Malaysia, in 1936 [13]. Chiggers and rodents are thought to be most important reservoirs of scrub typhus and murine typhus infection, respectively. Studies suggest that scrub typhus is more common in rural areas and murine typhus in urban areas [11], [13], [14], [15], [16], [17], [18], [19] but there is very little recent information on the epidemiology of scrub and murine typhus in places where both diseases occur.

The Lao People's Democratic Republic (Laos) is situated mostly east of the Mekong River and borders Thailand, Cambodia, Burma, China and Vietnam. The majority of the population (88%) of 5.6 million people lives in rural areas (2005 census from National Statistics Centre). Vientiane, the capital of Lao PDR is the most populated urban area in the country, with less than 300,000 inhabitants.

The diagnosis of non-malarial fevers in Laos remains difficult due to limited laboratory diagnostic facilities. In 2000, the main differential diagnoses for adults admitted with fever to hospital were slide-positive malaria or slide-negative ‘malaria syndrome’ and, in both situations patients were treated with antimalarials, with additional antibiotics for those with ‘malaria syndrome’. Since then it has become apparent that both scrub and murine typhus are common causes of fever in Laos [20], [21], [22]. In Mahosot Hospital, Vientiane, among 427 adults admitted with unexplained fever, 14.8% and 9.6% had serological evidence for scrub typhus and murine typhus, respectively [20]. As these diseases are usually relatively straightforward and inexpensive to treat with short courses of doxycyline, their recognition in Laos raises the prospect that a significant proportion of non-malarial fevers can be diagnosed and treated relatively inexpensively.

However, there is very little recent information on the epidemiology of scrub typhus and murine typhus especially where they are sympatric, as is the case in Vientiane, and there is a need for greater understanding of contrasting risk factors involved in scrub and murine typhus transmission. Therefore, we analyzed serological data from a randomly selected population of adults living in different neighbourhoods in Vientiane to determine the frequency of IgG seropositivity against scrub and murine typhus as indices of prior exposure to these pathogens.

## Materials and Methods

The research program entitled “Urbanization, Governance and Spatial Disparities of Health in Vientiane” aimed at describing and analysing the organization of urban areas (resulting from geographical, social, cultural, political, environmental, and behavioural combinations) as sources of intra-urban health inequalities. To provide health data on population of Vientiane, the Institut de Recherche pour le Développement and University Paris Ouest La Defense carried out a health survey in which indicators of health status and health seeking behaviour were studied [23], [24].

### Sample design and studied population

Vientiane Capital City (VCC) refers to the province that includes the Vientiane urban agglomeration, as well as surrounding smaller urban areas and rural villages [25]. Urban Vientiane only was estimated to have 277,000 inhabitants in 2005. This urban area is composed of 148 villages (‘ban’ in Lao), which are the primary administrative units and constituted the primary sampling unit of this seroprevalence survey. To define level of urbanization of neighbourhood in Vientiane city, we used some indicators based on 1995 and 2005 census and a variety of GIS-based indicators derived from 1999 aerial photographic coverage [26] rather than a single common indicator like population density. We selected thirteen indicators: built-up density, density of population, changes in built-on surface area between 1981 and 1999, proportion of public infrastructure buildings, proportion of trade buildings, number of markets in proximity, distance to the city centre via the road network, average distance of every building to the road network, access to running water, electricity and toilets, proportion of concrete houses, and proportion of the population involved with agricultural activities. Using a prior hierarchical classification, three categories of neighborhoods in Vientiane were identified: 1) the central zone; 2) the first urbanized belt; and 3) the second urbanized belt, with respectively 25, 67 and 56 neighbourhoods in each area. The first urbanized belt clearly differed from the central zone by a smaller proportion of public infrastructures, trade buildings and concrete houses. The first urbanized belt differed from the second belt by a higher density of built-up and of population and by household facilities (such as running water, electricity and modern toilettes) of much better quality.

To carry out the seroprevalence survey, nine neighborhoods were selected in each urban category (i.e. a total of 27 neighborhoods) as representative of the variability of the urban population. In each neighborhood, households were selected randomly from a list of households. Within each selected household, one adult (≥35 years) was randomly selected. To measure the association of place of residence with typhus seroprevalence rates, study participants were limited to adults who claimed continued residence in a single village of the study area for a minimum of five years. The population survey techniques have been described elsewhere [23], [24].

### Ethics statement

Ethical approval for the study was granted by the Lao National Ethics Committee for Health Research in Lao PDR (No 046) and the Oxford University Tropical Research Ethics Committee (OXTREC 003-06). All participants gave informed written consent prior to survey administration and sample collection.

### Household and individual survey

The survey took place in February and March 2006. Standardized questionnaires were administered to assess demographic and socioeconomic information from the study population. Individual information about sex, age, origin, education level, occupation, length of residence in Vientiane city, contact with rats were collected. Information about household size and the presence of rats around the house and the sanitary condition of the household plot (presence of rubbish and animal excrement) were also recorded. A household deprivation index was developed from household characteristics (e.g. house building materials, access to running water, types of cooking energy, possession of motorbike, car, refrigerator, washing machine and computer). Using Multiple Correspondence Analysis followed by Hierarchical Ascendant Classification, households incomes were classified in three categories: household with poor income (11% of sampled population), with intermediate income (61%) and those with high income (28%). The distance from the house to the closest market was measured in a Geographical Information System and the density of buildings in residential neighborhood was also calculated from data derived from 1999 aerial photographic coverage [26]. This density - which ranged from 5 to 100% - was grouped into tertiles (with thresholds of 65% and 84%) and classified as low, intermediate and high density. For every surveyed adult, blood sample of approximately 75 µl were collected by fingerpick, absorbed on Proteinsaver™ filter papers (Whatman plc, Maidstone, UK) and stored at −80°C until use.

### ELISA assays

Two discs of six mm diameter were cut, using a hole punch, from the centre of the dried filter paper blood spot and eluted at 37°C overnight in 500 µl of phosphate buffered saline (PBS) corresponding to a 1/25 dilution of original serum. The Rickettsia Scrub Typhus Group IgG ELISA (E-RST01G, PanBio Diagnostics, Brisbane, Australia) was used for the detection of anti-*O. tsutsugamushi* IgG antibodies and the manufacturers instructions were followed [27], [28].

Anti-*R. typhi* antibody detection used an in house typhus group IgG ELISA technique [17]. In brief, one half of each 96 well microtiter plate was coated with 100 µl/well of *R. typhi* (Wilmington) whole cell antigen (1∶3000 dilution) and 100 µl PBS was added per well to those in the other half of the plate. The plates were covered with a plastic lid and stored at +4−8°C for 2 days, washed 3 times with wash buffer (0.1% Tween 20 in PBS), blocked with 5% skim milk (Cadbury, Bournville, Worcs., UK) in wash buffer (dilution buffer) and incubated at 37°C for one hour. Filter paper elutes were diluted with dilution buffer to a working concentration of 1∶100, transferred to the plates and incubated at room temperature for 1 hour followed by 5 washes with wash buffer. The wells were incubated with an HRP _abelled affinity-purified antibody to human IgG (H+L) (KPL, Maryland, USA) at a dilution of 1∶2000 for one hour at room temperature. After washing 5 times, 100 µl/well of a peroxidase substrate, 2,2-azino-di-[ethylbenzthiazoline sulfonate] (ABTS)(KPL) was added and the plate incubated in the dark for 30 minutes at room temperature. 100 µl/well of ABTS stop solution (KPL) was added and the plate read immediately using a Multiskan EX ELISA reader (Labsystems, MA, USA) at 405 nm. The same ELISA plate reader was used to measure absorbance for scrub typhus group IgG assays. Equivocal results in both tests were repeated once. If the repeat test result remained as equivocal it was considered as negative in the statistical analysis.

To determine the concordance of the two ELISA techniques using sera and filterpaper bloodspot elutes, both ELISA techniques were performed on these samples collected as a part of the study of Phetsouvanh *et al.* [21] from the same patients at the same time point.

### Statistical analysis

Proportions and 95% Confidence Intervals (CI) were calculated, taking into account the two-stage sample design. Differences in seropositivity between areas were calculated by the Pearson chi-squared test. Potential factors associated with scrub typhus and murine typhus past exposure were explored first using a bivariate analysis and, secondly, using multivariate logistic regression. Multivariate logistic regressions were performed using Intercooled Stata 10 (Stata Corporation, College Station, Tx, USA) with fitting of a random-effect logit model at the neighborhood scale. Odds Ratios (OR) and 95% CI were calculated. Only finals models of multivariate logistic regression with significant risk factors (and with adjustment on age and sex) were presented in this paper. A *p* value of <0.05 was considered as significant. Maps were generated using Geographic Information System (GIS) ArcGis 9.2 software (ESRI, USA).

## Results

### Description of the surveyed population

A sample of 2,002 adults was included in the study with a mean age (range) of 50.6 (35–90) years. Only 9% of population was excluded from the surveyed sample because of a length of residence less than five years. The population was older in the central zone than in the first or second urbanized belt with 18%, 16% and 13% of the population aged >65 years, respectively (Table 1). The sex ratios did not statistically differ (p = 0.26) within the city, although the proportion of women was slightly higher in the central zone (63%) compared to the rest of the city (58%). The proportion of non-Lao people was slightly - but not statistically (p = 0.08) - higher (8%) in the central zone compared to the rest of the city (5%). The population living in the second urbanized belt had a significant lower education level than those living in the central zone or first urbanized belt since 30% versus 40%, respectively, attended secondary school. Income of sampled households varied by the extent of urbanization: households with high income were much more frequent in the central zone and in the first urbanized belt (33% and 31% respectively) than in the second urbanized belt (18%). Forty-three percent of sampled adults had lived in Vientiane for more than two thirds of their lifetime, without significant variation (p = 0.09) across the city.

**Table 1:** Surveyed population of Vientiane city according to their place of residence.

Category	Sub category	Overall city; n = 2,002	Central zone; n = 667	1^rst^ urbanized belt; n = 666	2^nd^ urbanized belt; n = 669	*Pearson test (p)*
**Sex**	Female *(/male)*	1,204 (59.0)	416 (62.7)	398 (57.6)	390 (57.8)	>0.05
**Age (years)**	35–44	775 (39.0)	224 (33.8)	244 (34.9)	307 (46.2)	
	45–54	534 (26.5)	182 (27.0)	171 (26.2)	181 (26.4)	**<0.05**
	55–64	381 (19.0)	141 (21.2)	144 (22.6)	96 (14.3)	
	≥65	312 (15.5)	120 (18.0)	107 (16.3)	85 (13.1)	
**Origin**	Non-Lao *(/Lao)*	123 (5.8)	59 (8.3)	30 (5.1)	34 (5.8)	>0.05
**Education**	No education	217 (11.2)	78 (11.8)	66 (11.4)	73 (10.7)	
	Primary school	1,058 (52.5)	342 (50.9)	311 (46.8)	405 (58.8)	**<0.05**
	Secondary school up	727 (36.3)	247 (37.3)	289 (41.8)	191 (30.5)	
**Occupation** 1	Farmer	47 (2.4)	7 (1.0)	7 (0.9)	33 (4.7)	
	Manual worker	160 (4.7)	28 (4.7)	41 (6.5)	91 (12.9)	<0.05
	Office worker	520 (28.0)	161 (24.4)	213 (35.7)	146 (23.1)	
	Retail traders, artisan…	577 (26.4)	243 (35.6)	156 (19.9)	178 (26.4)	
	Manager	90 (4.6)	35 (4.9)	42 (6.7)	13 (2.5)	
	Not working. At home	577 (28.5)	180 (27.3)	200 (29.3)	197 (28.7)	
**Household income** 2	Low	197 (9.9)	52 (8.0)	34 (4.4)	111 (16.1)	
	Middle	1,228 (63.4)	383 (58.9)	396 (64.1)	449 (65.8)	**<0.05**
	High	577 (26.7)	232 (33.0)	236 (31.5)	109 (18.1)	
**Length of residence in Vientiane**	Lifetime >2/3rds *(/<2/3rds)*	896 (42.7)	327 (47.4)	274 (38.7)	295 (43.3)	>0.05

1 *occupation current or the last one for those who have retired.*

2 *index of household income was developed with Multiple Correspondence Analysis followed by Hierarchical Ascendant Classification from several household characteristics (e.g. house building materials, access to running water, types of cooking energy, possession of motorbike, car, refrigerator, washing machine and computer).*

*Note: Proportions were performed taking into account the two-stage of sample design.*

### Comparison of ELISAs using sera and filterpaper elutes

Comparison of anti-scrub typhus IgG ELISA assays using 47 sera and filterpaper bloodspot pairs demonstrated agreement (i.e. positive or negative for IgG against *O. tsutsugamushi*) for 45 (96%) – one pair was negative for IgG from filterpaper but positive from serum and one pair positive for IgG from filterpaper but negative from serum. Comparison for anti-murine typhus IgG ELISA assays using 45 sera and filterpaper bloodspot pairs demonstrated agreement for 42 (93%) – two pairs were negative for IgG from filterpaper but positive from serum and one pair positive for IgG from filterpaper but negative from serum. Therefore, these anti-typhus IgG ELISAs using filterpaper elutes gave good agreement with results obtaining using sera.

### Overall seroprevalence

The overall percentage of scrub typhus IgG antibodies was 20.3% (CI = 18.1–22.5) and 20.6% (CI = 17.4–23.8) for murine typhus IgG antibodies (Table 2). Four percent of samples had IgG antibodies against both scrub and murine typhus.

**Table 2:** Percentage of inhabitants with IgG antibodies against scrub typhus and murine typhus (Vientiane city, 2006).

IgG antibody reacting sera (n = 2,002)	Result	Total (%)	95% CI
**Scrub Typhus**	Positive	394 (20.3)	18.1–22.5
	Negative	1488 (73.7)	70.7–76.8
	Equivocal	120 (5.9)	4.6–7.2
**Murine Typhus**	Positive	440 (20.6)	17.4–23.8
	Negative	1562 (79.4)	76.2–82.6
**Scrub + Murine Typhus**	Positive	80 (3.6)	2.5–4.7
	Negative	1248 (62.7)	59.6–65.7
	Mixed	674 (33.7)	31.3–36.2

*Note: Proportions and their 95% confidence intervals (CI) were performed taking into account the two-stage of sample design.*

### Spatial distribution of seroprevalence

**Figure 1:**
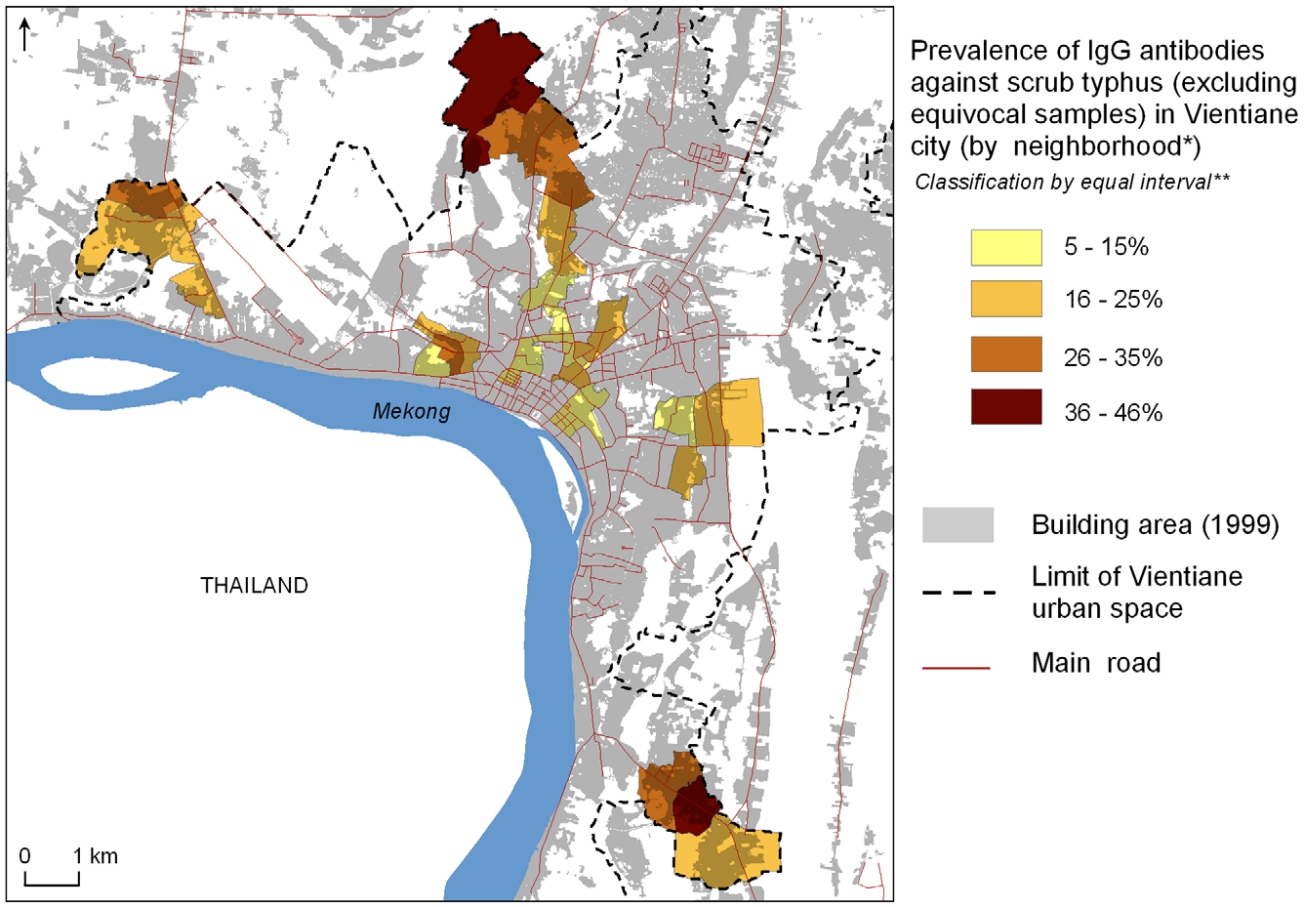
Spatial distribution of households with a member IgG positive against scrub typhus in Vientiane city in 2006. * The « neighborhood » is the primary administrative unit in Laos and constituted the primary sampling unit of the seroprevalence survey within Vientiane. ** Equal Interval Classification method divides a set of attribute values into groups that contain an equal range of values. Note: Cartographic files from “Atlas Infographique de Vientiane” [26].

**Figure 2:**
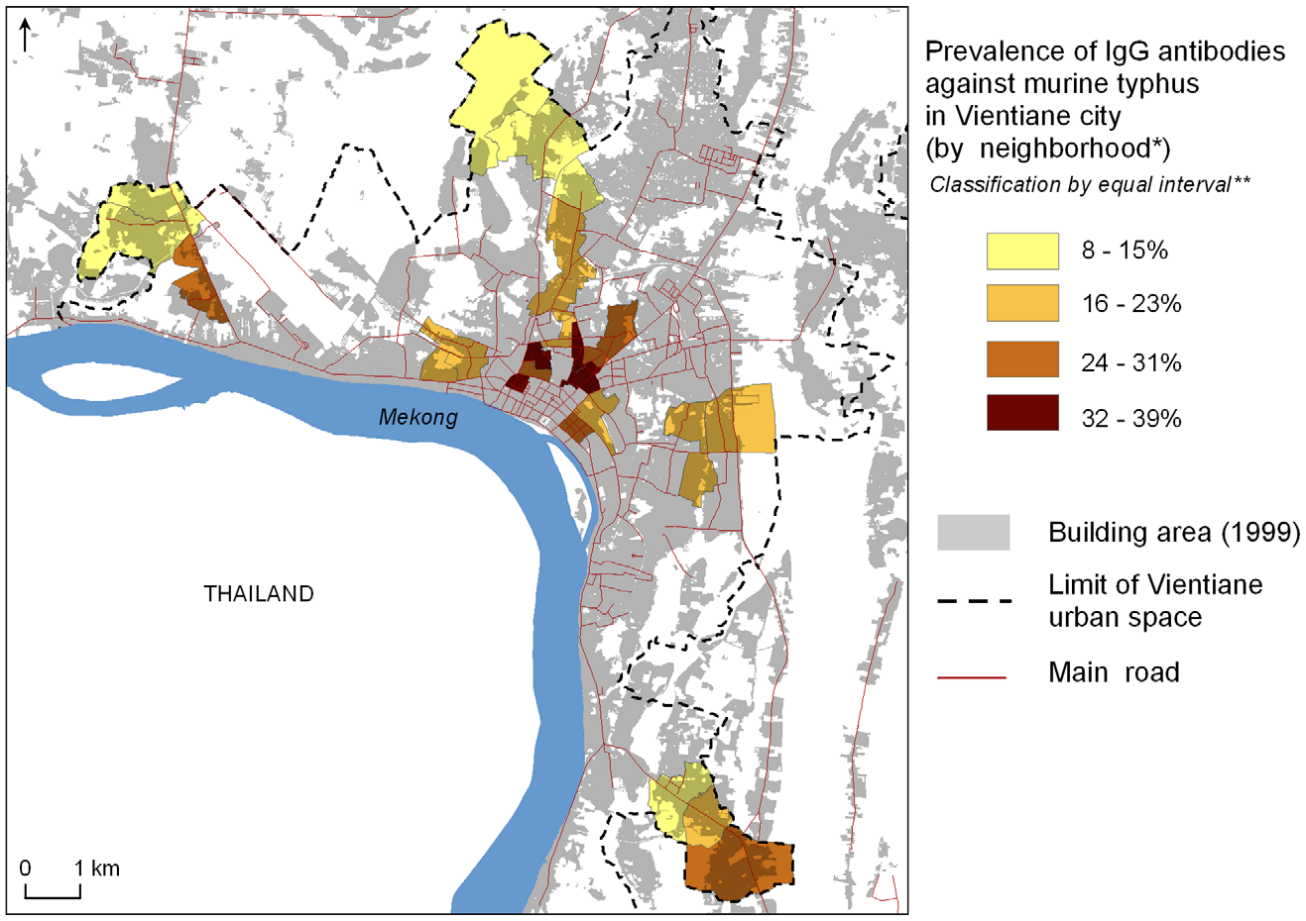
Spatial distribution of households with a member IgG positive against murine typhus in Vientiane city in 2006. * The « neighborhood » is the primary administrative unit in Laos and constituted the primary sampling unit of the seroprevalence survey within Vientiane. ** Equal Interval Classification method divides a set of attribute values into groups that contain an equal range of values. Note: Cartographic files from “Atlas Infographique de Vientiane”[26].

The prevalence of scrub typhus IgG antibodies was significantly higher (p<0.01) among people living in the periphery than in the central zone: 13.1% positive in the central zone as compared to 16.8% and 28.4% for first and second urbanized belts, respectively (Table 3, Figure 1). In contrast, seroprevalence of murine typhus IgG antibodies was significantly higher (p<0.01) in the central zone (30.8%) as compared to the first (20.1%) and second (14.4%) urbanized belts (Table 3, Figure 2). Two villages (*Bonangua* and *Somvang Tay*) located in the extreme north and extreme southeast of the city had very high seroprevalence of scrub typhus, exceeding 35% (Figure 1). Highest seroprevalences (>32%) against murine typhus occurred in four villages (*Anou*, *Thongkhankham Neua*, *Sisavat Tay* and *Hatsadi Neua*).

**Table 3:** Bivariate analysis of factors associated with IgG positivity against scrub and murine typhus (Vientiane city, 2006).

		Scrub Typhus^1^		Murine Typhus	
Category	Sub category	Number Positive/Tested (%)	Pearson test (p)	Number Positive/Tested (%)	Pearson test (p)
**Sex**	Male	143/798 (18.2)		164/798 (19.1)	
	Female	251/1204 (21.8)	>0.05	276/1204 (21.6)	>0.05
**Age (years)**	35–44	105/775 (13.6)		157/775 (18.8)	
	45–54	89/534 (17.9)	**<0.01**	127/534 (22.3)	>0.05
	55–64	99/381 (26.4)		88/381 (23.0)	
	≥65	101/312 (34.3)		68/312 (19.1)	
**Origin**	Lao	371/1879 (20.4)		413/1879 (20.7)	
	Non-Lao	23/123 (19.0)	>0.05	27/123 (18.9)	>0.05
**Education**	No education	71/217 (34.5)		46/217 (19.4)	
	Primary school	228/1058 (22.4)	**<0.01**	230/1058 (20.5)	>0.05
	Secondary school up	95/727 (13.01)		164/727 (21.1)	
**Occupation** 2	Farmer	24/47 (48.5)		5/47 (7.9)	
	Manual worker	35/160 (22.6)	**<0.05**	29/160 (18.9)	>0.05
	Office worker	100/520 (18.3)		106/520 (19.1)	
	Retail traders, artisan…	86/577 (15.3)		138/577 (23.0)	
	Manager	17/90 (20.9)		16/90 (15.7)	
	Not working. At home	122/577 (23.2)		139/577 −22.1)	
**Worked rice fields in the past 12 months**	Yes	31/81 (34.8)		9/81 (10.2)	
	No	362/1919 (19.7)	**<0.01**	430/1919 (21.0)	**<0.01**
**Length of residence in Vientiane city**	<1/3rds lifetime	58/160 (35.4)		21/160 (11.7)	
	1/3–2/3rds lifetime	163/780 (21.7)	**<0.01**	167/780 (21.1)	**0.03**
	>2/3rds lifetime	144/896 (16.3)		206/896 (20.9)	
**Contact with rats (by touching)**	Yes	28/87 (33.6)		19/87 (20.7)	
	No	366/1915 (19.6)	**<0.01**	421/1915 (20.6)	>0.05
**Household income** 3	Low	58/197 (29.2)		37/197 (18.7)	
	Middle	252/1228 (21.2)	**<0.01**	281/1228 (21.2)	>0.05
	High	84/577 (15.0)		122/577 (19.8)	
**Household size**	Small (≤3 people)	36/224 (16.2)		43/224 (16.9)	
	Intermediate (4–6 people)	201/1109 (18.8)	**<0.01**	245/1109 (20.8)	>0.05
	Large (≥7 people)	157/669 (24.1)		152/669 (21.3)	
**Plot sanitary condition** 4	Clean	339/1809 (19.5)		396/1809 (20.4)	
	Not clean	55/193 (28.4)	**<0.05**	44/193 (22.6)	>0.05
**Distance from house to closest market**	Very close (<300 m)	61/398 (17.0)		91/398 (22.9)	
	Close (300–500 m)	110/608 (18.5)	>0.05	145/608 (22.1)	>0.05
	Middle (500–750 m)	99/488 (21.0)		122/488 (22.7)	
	Far (>750 m)	124/508 (24.0)		82/508 (15.3)	
**Rats seen in house or garden**	Yes	260/1338 (20.3)		304/1338 (21.1)	
	No	134/664 (20.5)	>0.05	136/664 (19.6)	>0.05
**Urbanization level**	2^nd^ Belt	203/669 (28.4)		101/669 (14.4)	
	1rst Urbanized Belt	106/666 (16.8)	**<0.01**	134/666 (20.1)	**<0.01**
	Central Zone	85/667 (13.1)		205/667 (30.8)	
**Built-up density** 5	Low (≤65%)	172/659 (25.3)		108/659 (15.5)	
	Middle (65–85%)	136/667 (20.5)	**<0.01**	135/667 (19.6)	**<0.05**
	High (≥85%)	86/676 (13.6)		197/676 (28.4)	

1 *excluding equivocal samples.*

2 *current occupation or last occupation for elderly.*

3 *index of household income was developed from several household characteristics (e.g. house building materials, access to running water, types of cooking energy, possession of motorbike, car, refrigerator, washing machine and computer).*

4 *the sanitary condition of the household plot of land (presence of rubbish, animal excrement, etc.) was assessed by investigators.*

5 *built-up density reflects the proportion of the neighborhood land area covered by building.*

*Note: Proportions were performed taking into account the two-stage of sample design.*

### Risk factors for scrub typhus past exposure

**Figure 3:**
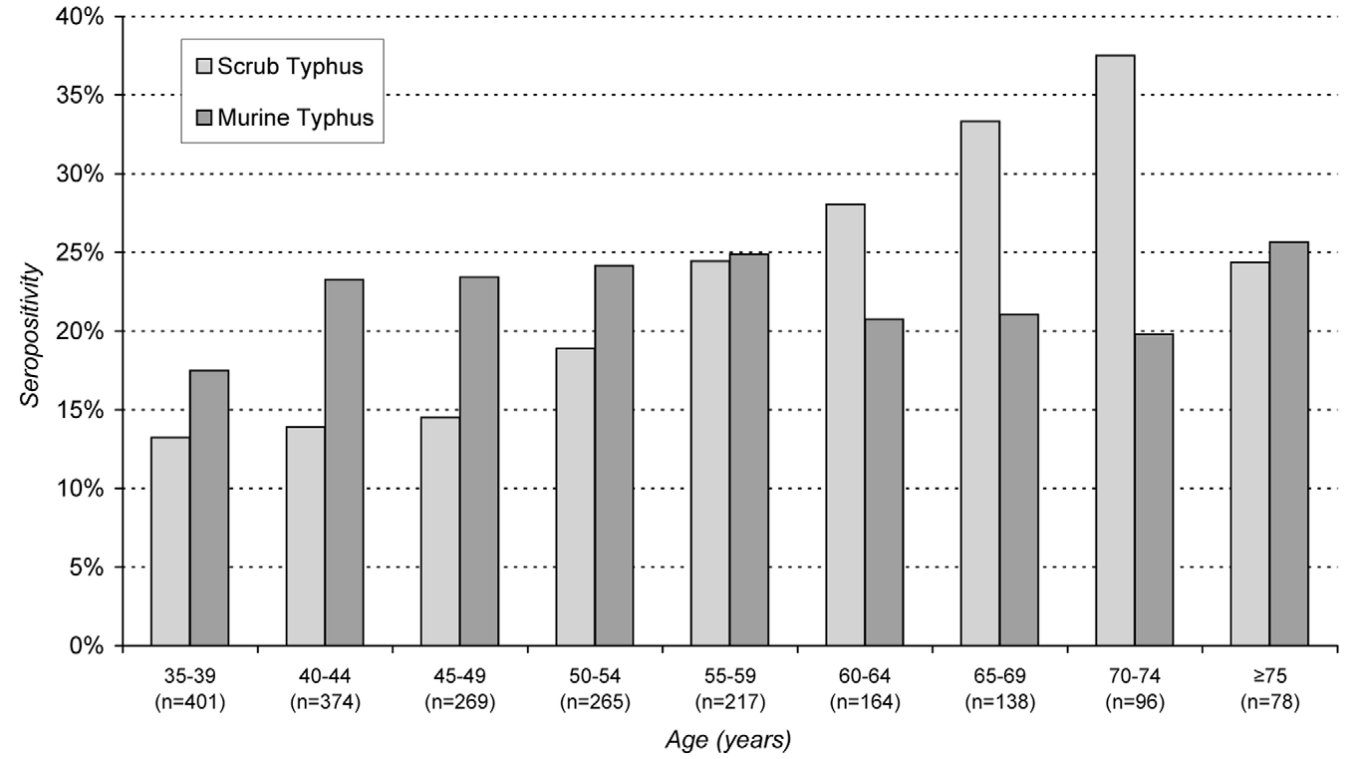
Percentage IgG positivity against scrub typhus and murine typhus among adults (aged >35 years) of different age (in 5 years age classes), Vientiane city, 2006.

Numerous individual (age, education level, occupation, length or residence in Vientiane, tactile contact with rats), household (income, size and sanitary condition) and neighbourhood characteristics (level of urbanization and density of buildings) were statistically associated in bivariate analysis with IgG antibodies against scrub typhus (Table 3). The older the population, the higher was the prevalence of scrub typhus seropositivity (Figure 3).

In multivariate analysis, eleven characteristics remained significantly associated with IgG antibodies against scrub typhus. Being female, >55 years old, a farmer, Lao citizen, having had no education, had tactile contact with rats, living in households with low income, in larger households (>4 people), in plots of land with poor sanitary conditions, living in neighborhoods with a low building density and having lived in Vientiane City for less than one third of lifetime increased significantly the risk for past infection with scrub typhus (Table 4).

**Table 4:** Multivariate logistic regression analysis of factors associated with IgG positivity against scrub typhus (Vientiane city, 2006).

Category	Sub category	Positive^1^ Odd Ratio [IC 95%]	p value
**Sex**	*Male*	*Reference*	
	Female	1.5 [1.1–1.9]	**<0.01**
**Age (years)**	*35–44*	*Reference*	
	45–54	1.3 [0.9–2.0]	**<0.01**
	55–64	2.6 [1.8–3.9]	
	≥65	3.6 [2.4–5.2]	
**Origin**	*Non-Lao*	*Reference*	
	Lao	1.9 [1.1–3.4]	**0.02**
**Education**	*Secondary school and above*	*Reference*	
	Primary school	1.3 [0.9–1.7]	**0.04**
	No education	1.5 [1.0–2.3]	
**Occupation** 2	*Non Farmer*	*Reference*	
	Farmer	2.1 [1.0–4.2]	**0.04**
**Length of residence in Vientiane**	*>2/3rds lifetime*	*Reference*	
	1/3–2/3rds lifetime	1.5 [1.1–2.0]	**0.02**
	<1/3rds lifetime	2.2 [1.3–3.9]	
**Contact with rats (by touching)**	*No*	*Reference*	
	Yes	2.4 [1.6–3.4]	**<0.01**
**Household income** 3	*High*	*Reference*	
	Middle	1.4 [0.9–2.2]	**<0.01**
	Low	2.3 [1.4–3.9]	
**Household size**	*Small (<4 people)*	*Reference*	
	Large (≥4 people)	1.9 [1.2–3.1]	**<0.01**
**Plot sanitary condition** 4	*Clean*	*Reference*	
	Not clean	1.7 [1.1–2.7]	**0.02**
**Building density** 5	*High (≥85%)*	*Reference*	
	Intermediate (65–85%)	1.7 [1.2–2.4]	**<0.01**
	Low (≤65%)	2.3 [1.6–3.3]	

1 *excluding equivocal samples.*

2 *current occupation or last occupation for elderly.*

3 *index of household income was developed from several household characteristics (e.g. house building materials, access to running water, types of cooking energy, possession of motorbike, car, refrigerator, washing machine and computer).*

4 *the sanitary condition of the household plot of land (presence of rubbish, animal excrement, etc.) was assessed by investigators.*

5 *built-up density reflects the proportion of the neighborhood land area covered by building.*

### Risk factors for murine typhus past exposure

Factors associated in bivariate analysis with the presence of IgG antibodies against murine typhus were less frequent: longer residence in Vientiane, absence of recent labour in rice fields, living in central urban zone and in neighbourhoods with higher building density (Table 3). In multivariate analysis (Table 5), four characteristics were significantly associated with IgG antibodies against murine typhus - living in Vientiane for more than one third of their lifetime, being a retail trader, staying at home or not working, living close to markets (<750 m) and in neighborhoods with high building density increased significantly the risk for past infection with murine typhus.

**Table 5:** Multivariate logistic regression analysis of factors associated with IgG positivity against murine typhus (Vientiane city, 2006).

Category	Sub category	Positive Odd Ratio [IC 95%]	p value
**Sex**	*Male*	*Reference*	
	Female	1.0 [0.8–1.3]	>0.10
**Age (years)**	*35–44*	*Reference*	
	45–54	1.2 [0.9–1.7]	>0.10
	55–64	1.1 [0.7–1.8]	
	≥65	1.1 [0.7–1.5]	
**Length of residence in Vientiane**	*<1/3rds*	*Reference*	
	>1/3rds	1.7 [1.1–2.7]	**0.02**
**Occupation** 1	*Farmer, manual and office worker, Manager*	*Reference*	
	Retail traders/Non- working/At home	1.4 [1.1–1.7]	**0.02**
**House/Market distance**	*Long* (>750 m)	*Reference*	
	Short (<750 m)	1.5 [1.1–2.2]	**0.02**
**Building density** 2	*Low* 2 *(≤65%)*	*Reference*	
	Intermediate (65–85%)	1.2 [0.7–2.1]	**0.01**
	High (≥85%)	1.9 [1.2–3.1]	

1 *current occupation or last occupation for elderly.*

2 *built-up density reflects the proportion of the neighborhood land area covered by building.*

### Risk factors for both murine typhus and scrub typhus past exposure

In multivariate analysis (table not presented) people aged >55 years (OR = 2.5; 95%CI = 1.3–4.8; p<0.01), those from a poor household (OR = 2.3; 95%CI = 1.0–5.3; p = 0.05) and those living in plots of land with poor sanitary conditions (OR = 1.8; 95%CI = 1.0–3.5; p = 0.05) were at greater risk of having IgG antibodies against both scrub and murine typhus. None of the neighborhood factors were associated with the concurrent presence of IgG antibodies against both scrub and murine typhus.

## Discussion

This study examined the presence of IgG antibodies as surrogate markers for past infection with agents known to cause two common rickettsial diseases in and around an Asian city. Patients with both diseases present at Vientiane health facilities and are sympatric at the district level [20]. However, adults living in central, more urbanised area of Vientiane had a higher seropositivity against murine typhus and, adults living in peripheral less urbanised Vientiane had a higher seropositivity against scrub typhus. This confirms that those living in more rural areas are at higher risk of scrub typhus infection and, those living in urban areas are at more risk of murine typhus infection, which is consistent with what has been observed elsewhere [11], [12], [15], [16], [17], [18], [19]. The absence of previous serological surveys does not allow examination of temporal changes in transmission of these diseases, which were first described in Laos in 2006 [20]. In a paper resulting from the same survey, variation according to the level of urbanization was also noticed for the spatial distribution of flavivirus exposure in Vientiane city with anti-flavirus IgG prevalence significantly higher among individuals living in the central city (60.1%) than those living in the periphery (44.3%) [29].

There are at least three possible explanations for apparent urban scrub typhus in Vientiane. Urban inhabitants may have acquired the infection in prior rural residence elsewhere, in visits to rural areas to help with farming, collecting bamboo shoots, hunting and fishing or they could have contracted the infection in urban areas. All are likely to be important. Anecdotally, a significant minority of patients admitted with scrub typhus to Mahosot Hospital, Vientiane, had been to rural areas during the putative incubation period (∼7–10 days) to help with their families' farming (RP, PN) but the disease could also be contracted in parks, fields and gardens within the city. In part because of the terrible toll *O. tsutsugamushi* took on troops in scrublands in Burma & NE India in the Second World War it came to be known as scrub typhus. However, contrary to what textbooks still claim [30] scrub typhus also commonly occurs in palm plantations, primary forest, beaches, gardens [14], [31] and also from metropolitan areas as Bombay (Mumbai) [32], Jakarta [16], [33], [34], suburban Bangkok [35], [36], Komatsu City, Japan [37], Yuxi City, China [19] and Calcutta [38]. In view of the broader ecological distribution than is implied by the term ‘scrub typhus’, the original Japanese name of tsutsugamushi, as suggested by Cadigan *et al.* [14], may be less confusing.

Richards *et al.* [17] examined the seroepidemiology of sympatric murine and scrub typhus in Java through a cross-sectional community-based survey in rural, suburban and urban areas in Malang District. They found prevalences of anti-*O. tsutsugamushi* IgG and anti-*R. typhi* IgG of 1.3% and 34.7%, respectively. Amongst, presumably urban, Kuala Lumpur blood donors, Tay *et al.* [18] found that 5.4% and 9.2% were seropositive against *O. tsutsugamushi* and *R. typhi*, respectively. They suggested that ‘with rapid economic development…the close proximity of agricultural habitats and urban development may allow the transmission of rickettsial diseases in the urban areas’ [18]. The results from Vientiane contrast with those from Malang and Kuala Lumpur in that many more inhabitants of Vientiane had evidence for prior exposure to scrub typhus. Without comparable data on rural exposure and length of residence in urban areas it is difficult to interpret this difference.

Indices of poverty, such as level of education, farming, low household income and poor plot sanitary conditions were studied in relation with past exposure to scrub and murine typhus. Although these indices are not independent, it is interesting to note that they are all significantly associated with past exposure to scrub typhus in multivariate analysis. However, it is not the case for past exposure to murine typhus since, of variables potentially linked to poverty, only occupation appears as a significant variable. Different aspects of poverty may then be involved in risks to exposure to scrub or murine typhus. More qualitative research is needed to evaluate relationship between poverty and typhus transmission.

That non-Lao people are at less risk to previous exposure to scrub typhus may reflect the fact that they are less involved in familial farming activities and that they are preferentially living in more urbanised neighbourhoods [23]. Why women should be at apparent increased risk to have been exposed to scrub typhus is unclear as both sexes participate in farming. The probability of having positive IgG antibodies against scrub typhus clearly increased with age. However, we did not observe any significant relationship between age and seropositivity for murine typhus. Adults with long residency in Vientiane had a higher frequency of IgG antibodies against murine typhus. As rodent and flea densities are likely to be higher in urban settings, people living for a long time in Vientiane may be more exposed to murine typhus. This is supported by the association of IgG against murine typhus with higher building density and closeness to markets and is reflected in the old name of ‘shop typhus’ for murine typhus.

Limitations of this study include that we did not collect information about other places visited outside residential neighbourhood (i.e. place of work, leisure, travel) or former places of residence, and that we used old land use coverage data whilst the Vientiane landscape has changed considerably since these land use data were collected in 1999. In addition only one person was sampled in each household, which did not allowed for examination of clustering within households. Furthermore we surveyed only adults >35 years old and could therefore not examine changes in seroprevalence in younger people, in whom a significant proportion of seroconversions are likely to occur [16], [17]. It is unknown what proportion of patients with IgG against these two rickettsial agents developed disease. Surprisingly, there are few data on the longevity of IgG, on locally appropriate cut offs indicating past infection or of the consequences of repeated infections on antibody titres against *O. tsutsugamushi* and none that we are aware of for IgG against *R. typhi*. Saunders *et al*. [39] calculated that the annual reversion rate for antibodies against *O. tsutsugamushi* to a titre <l:50 was 6l%, suggesting that our data are likely to underestimate the true frequency of past infections. Hence, there remains uncertainty as to the most appropriate diagnostic techniques and correct serology cutoff titres in different environments in the diagnosis of prior rickettsial infections in the healthy. The presence of IgG against these pathogens should be regarded as indices of exposure. Some patients may develop specific IgG without developing disease and some will lose their specific IgG during their lifetime. More research is needed to define antibody responses, against both pathogens, and their changes through time to define both acute disease [40] and past exposure. Possible cross-reactivity of the murine typhus IgG ELISA with other rickettsial pathogens is not entirely excluded but the most likely candidate for this, *R. prowazekii*, has not been recorded from Laos.

Vientiane is a rapidly growing city, with the population having almost doubled during the past 15–20 years [25]. As a result, the territory of the city has expanded into the paddy fields of its former rural hinterland and the city has embarked on a far-reaching path of urban transformation. The transformation in physical landscape and in way of life may have and will lead to a modification of scrub and murine transmission.

Both scrub and murine typhus tend to afflict the poorer citizens of Vientiane and are responsible for high incidence of undifferentiated fever. As they are relatively simple and inexpensive to treat, oral doxycycline may be an appropriate empirical therapy for those for without access to confirmatory tests. However, it would need to be borne in mind that other common diseases, from which rickettsial diseases are difficult to distinguish clinically, such as typhoid and dengue, would not respond to such therapy.

Public education campaigns on disease avoidance and chigger repellents, and community participation (rubbish disposal and rodent control, especially at markets) may reduce the incidence of scrub typhus and murine typhus [41], [42]. More knowledge is needed on the vectors and epidemiology of rickettsial diseases in urban Laos, especially whether scrub typhus-infected chiggers occur in urban areas and which flea and rodent species are involved in the local epidemiology of murine typhus.

## Supporting Information

Alternative Language Abstract S1 Translation of the abstract into French by Julie Vallée and Jean-Paul Gonzalez.
(0.02 MB DOC)
