# Supplementary material for: Contrasting Spatial Distribution and Risk Factors for Past Infection with Scrub Typhus and Murine Typhus in Vientiane City, Lao PDR
Source: PLoS Negl Trop Dis. 2010 Dec 7;4(12):e909. doi: 10.1371/journal.pntd.0000909 (PMC2998433; doi:10.1371/journal.pntd.0000909)
Supplement: Alternative Language Abstract S1 — Translation of the abstract into French by Julie Vallée and Jean-Paul Gonzalez. (0.02 MB DOC) [file pntd.0000909.s001.doc]

***en Français***

**TitRe**

Distribution spatiale et facteurs de risques de l’exposition passée au typhus des broussailles et au typhus murin dans la ville de Vientiane, Laos.

**Résumé**

**Contexte**

Le diagnostic étiologique des fièvres demeure difficile au Laos en raison des moyens de laboratoire limités. Toutefois, on a récemment constaté que le typhus des broussailles et le typhus murin constituaient des causes fréquentes de fièvres non diagnostiquées. Si les données épidémiologiques suggèrent que le typhus des broussailles serait plus fréquent dans les zones rurales et le typhus murin dans les zones urbaines, très peu d’information sont disponibles sur les facteurs de transmission du typhus des broussailles et du typhus murin dans les zones où ces maladies sont sympatriques, ce qui est le cas dans Vientiane, la capitale de la RDP Lao.

**Méthodologie et principaux résultats**

Nous avons alors calculé la fréquence en IgG positifs contre le typhus des broussailles (*Orientia tsutsugamushi*) et contre le typhus murin (*Rickettsia typhi*), comme autant d’indices d’une exposition passée à ces pathogènes, parmi une population d’adultes sélectionnés aléatoirement dans les espaces urbains et périurbains de Vientiane (n=2,002 ; ≥ 35 ans).

Les anticorps IgG du typhus des broussailles et du typhus murin ont été détectés par des tests ELISA à partir de papiers filtres élués. Nous avons validé la précision des éluâts pour ces tests ELISA en les comparant à ceux réalisés sur des échantillons de sérums connus.

La prévalence globale observée en anticorps IgG était de 20,3% pour le typhus des broussailles et de 20,6% pour le typhus murin. La séroprévalence IgG du typhus des broussailles s’avère significativement plus élevée parmi les adultes vivant en périphérie (28,4%) que parmi ceux des espaces centraux de la ville de Vientiane (13,1%). En revanche, la répartition de la séroprévalence IgG du typhus murin est significativement plus élevée dans les espaces centraux (30,8%) qu’en périphérie (14,4%).

A partir d’analyses statistiques multivariées, on observe que les adultes vivant depuis longtemps à Vientiane ont un risque significativement plus élevé d’avoir été en contact avec le typhus murin et un risque significativement plus faible d’avoir été en contact avec le typhus des broussailles. Les personnes qui ont un risque accru d’avoir été exposées au typhus des broussailles sont les agriculteurs, les personnes avec de faibles revenus et un faible niveau d’éducation, les membres des ménages nombreux et les personnes vivant sur des terrains peu salubres. Par ailleurs, les personnes vivant à proximité des marchés et dans des quartiers densément bâtis ont plus de risque d’avoir été en contact avec le typhus murin et moins de risque d’avoir été en contact avec le typhus des broussailles.

**Conclusions**

Cette étude souligne une intense circulation du typhus des broussailles et du typhus murin dans la ville de Vientiane. Elle met aussi en évidence la répartition spatiale contrastée des ces deux maladies et les différences des facteurs de risques impliqués dans leur transmission.
